# Mesothelin Expression in Triple Negative Breast Carcinomas Correlates Significantly with Basal-Like Phenotype, Distant Metastases and Decreased Survival

**DOI:** 10.1371/journal.pone.0114900

**Published:** 2014-12-15

**Authors:** Gary Tozbikian, Edi Brogi, Kyuichi Kadota, Jeffrey Catalano, Muzaffar Akram, Sujata Patil, Alice Y. Ho, Jorge S. Reis-Filho, Britta Weigelt, Larry Norton, Prasad S. Adusumilli, Hannah Yong Wen

**Affiliations:** 1 Department of Pathology, Memorial Sloan Kettering Cancer Center, New York, New York, United States of America; 2 Department of Surgery, Memorial Sloan Kettering Cancer Center, New York, New York, United States of America; 3 Department of Epidemiology and Biostatistics, Memorial Sloan Kettering Cancer Center, New York, New York, United States of America; 4 Department of Radiation Oncology, Memorial Sloan Kettering Cancer Center, New York, New York, United States of America; 5 Department of Medicine, Memorial Sloan Kettering Cancer Center, New York, New York, United States of America; 6 Center for Cell Engineering, Memorial Sloan Kettering Cancer Center, New York, New York, United States of America; University of North Carolina School of Medicine, United States of America

## Abstract

Mesothelin is a cell surface associated antigen expressed on mesothelial cells and in some malignant neoplasms. Mesothelin-targeted therapies are in phase I/II clinical trials. The clinicopathologic and prognostic significance of mesothelin expression in triple negative breast carcinomas (TNBC) has not been fully assessed. We evaluated the expression of mesothelin and of basal markers in tissue microarrays of 226 TNBC and 88 non-TNBC and assessed the clinicopathologic features of mesothelin-expressing breast carcinomas. Furthermore, we investigated the impact of mesothelin expression on the disease-free and overall survival of patients with TNBC. We found that mesothelin expression is significantly more frequent in TNBC than in non-TNBC (36% vs 16%, respectively; p = 0.0006), and is significantly correlated with immunoreactivity for basal keratins, but not for EGFR. Mesothelin-positive and mesothelin-negative TNBC were not significantly different by patients’ race, tumor size, histologic grade, tumor subtype, lymphovascular invasion and lymph node metastases. Patients with mesothelin-positive TNBC were older than patients with mesothelin-negative TNBC, developed more distant metastases with a shorter interval, and had significantly lower overall and disease-free survival. Based on our results, patients with mesothelin-positive TNBC could benefit from mesothelin-targeted therapies.

## Introduction

Mesothelin (MSLN) is a 40-kDa glycosylphosphatidylinositol-linked cell surface antigen present in normal mesothelial cells and overexpressed in several human malignancies, including mesothelioma, pancreatobiliriary, ovarian and lung adenocarcinomas [1]–[8]. In mesothelioma MSLN promotes tumor cell invasion by increased MMP-9 secretion [9]. MSLN also binds CA-125/MUC16 with very high affinity and may contribute to the adhesion of tumor cells in peritoneal metastasis [10], [11]. Mesothelin expression increases resistance to TNFα-induced apoptosis through Akt/PI3K/NF-κB activation and IL-6/Mcl-1 expression in pancreatic carcinoma cell lines [12]. MSLN-overexpressing pancreatic cancer cell lines showed increased cyclin E and cyclin dependent kinase 2 expression, resulting in increased cell proliferation and cell cycle progression [13]. Membrane-bound MSLN is also released into body fluids and its use as a potential serum tumor marker is currently under investigation [14], [15]. MSLN is an attractive target for targeted therapy due to its limited distribution in normal tissues, high immunogenicity, and elevated expression in several human malignancies [16]. Several ongoing clinical trials in patients with ovarian cancer, with pancreatic cancer or with mesothelioma suggest that MSLN-specific T-cell responses have a beneficial effect [16]–[22].

Triple negative breast carcinomas (TNBC) are invasive breast carcinomas that lack expression of estrogen receptor (ER), progesterone receptor (PR) and human epidermal growth factor receptor 2 (HER2). They constitute approximately 10–17% of all invasive breast carcinomas and tend to be more common in young women [23]–[28], and often of African-American or Hispanic ethnicity [27], [29], [30]. Patients with TNBC have an aggressive clinical course [23], [26]–[29], [31] characterized by short survival after the first metastatic event [26], [29] and death within 5 years of the initial diagnosis [26], [28]. Approximately 71–80% of TNBC are basal carcinomas by gene expression profiling [32]–[36]. Basal TNBC tend to have more aggressive clinical course than non-basal TNBC, with even earlier disease recurrence, often times with lung and/or brain metastases [31], [37]–[40], shorter disease free survival and breast cancer specific survival [41]. At present no effective targeted therapy is available for treatment of TNBC [42] and significant efforts are currently focused on the identification of novel therapeutic targets for these tumors.

In this study, we assessed the expression of MSLN in a large cohort of TNBC and non-TNBC. We also correlated MSLN overexpression with clinicopathologic features and basal-like immunophenotype of TNBC [39], [43]. Furthermore, we evaluated MSLN as a potential prognostic marker in TNBC by correlating its expression with clinical outcome.

## Materials and Methods

### Tissue microarrays

Tissue microarrays (TMAs) containing 226 TNBC and 88 non-TNBC were used in this study. A breast carcinoma was defined as TNBC if nuclear staining for ER and PR was detected in less than 1% of the tumor cells, and HER2 was negative (0 or 1+) by immunohistochemistry (IHC) or equivocal (2+) by IHC and showed no HER2 gene amplification by fluorescence in situ hybridization (FISH) [44], [45]. The TNBC cases were obtained from consecutive patients who underwent surgical excision of the primary breast carcinoma at our center between 2002 and 2006 and for which slides and blocks were available for the study. A TMA of non-TNBC from consecutive patients treated at our institution in 2004 was used for reference. Triplicate 0.6-mm diameter cores from formalin- fixed, paraffin-embedded blocks were used to construct the TMAs. Only carcinomas spanning 0.5 cm or larger were used for the TMAs, to ensure the availability of residual carcinoma for possible future clinical use. Tumor size, grade and the presence or absence of lymphovascular invasion (LVI) were extracted from the original pathology reports. Collection or study of existing data - application for exemption from IRB/PB review (including waiver of HIPAA authorization and informed consent) was reviewed and approved by the Institutional Review Board (IRB) and Human Biospecimen Utilization Committee.

### Immunohistochemistry

We assessed MSLN expression on TMAs by IHC (Vector Lab, USA, clone 5B2, 1∶50, mouse monoclonal Ab, lgG1). Mesothelioma tissue was used as positive control. Immunohistochemical stains for ER (Ventana Medical Systems, Inc. USA, clone 6F11, rabbit monoclonal Ab, lgG1), PR (Ventana Medical Systems, Inc. USA, clone 1E2, rabbit monoclonal Ab, lgG1), HER2 (Ventana Medical Systems, Inc. USA, clone 4B5, rabbit monoclonal Ab lgG1), and basal-like markers, including CK5/6 (Dako, clone D5/16B4, mouse monoclonal antibody, 1∶50 dilution), CK14 (AbCam, clone LL002, mouse monoclonal antibody, 1∶100 dilution), EGFR (Thermoscientific, clone EP38Y, rabbit monoclonal antibody, 1∶50 dilution) were performed with appropriate controls. Carcinomas showing any cytoplasmic staining for CK5/6 and CK14 were regarded as positive for these markers. Scoring of EGFR membranous reactivity followed the ASCO/CAP criteria for HER2 [45]; cases with EGFR staining intensity of 2+ or 3+ were regarded as positive. Two pathologists blinded to the patients’ clinical outcomes separately assessed MSLN expression in all TNBC and non-TNBC and concordant scores were obtained. A previously described [9] semi-quantitative scoring system was used to score MSLN reactivity. Briefly, the percentage of tumor cells with MSLN staining was assigned a score of 0 (<1% tumor cells), 1 (1%–50% tumor cells), or 2 (>50% tumor cells). Staining intensity was scored as 0 (none), 1 (weak), 2 (moderate) or 3 (strong). The final MSLN score was calculated by the sum of the percentage and intensity scores of each tumor. Any case with final MSLN score ≥3 was classified as positive. MSLN expression was correlated with age at diagnosis, tumor size, grade, LVI, regional lymph node involvement, subsequent distant metastases, interval to metastases, site of metastases, and survival.

### Statistical analysis

The relationship between MSLN staining, basal-like phenotype, and clinicopathologic features was assessed using Fisher’s exact test. Five-year estimates of overall survival (OS) and disease-free survival (DFS) by MSLN positivity, basal-like phenotype and clinicopathologic features were calculated using Kaplan-Meier methods. Differences between the Kaplan-Meier curves were tested using log-rank test. A p-value <0.05 was considered as statistically significant.

## Results

### Clinicopathologic features

Patients with TNBC and non-TNBC were similar in age. The mean age at diagnosis of patients with TNBC was 55 years (range, 54–57). The mean age of patients with non-TNBC was 54 years (range, 51–57). Among patients with TNBC, 163 (72%) were White, 48 (21%) were Black, 13 (6%) were Asian, and 2 (1%) were of other races. Among patients with non-TNBC, 75 (85%) were White, 10 (11%) were Black, 1 (1%) was Asian, and 2 (2%) were of other races. There was a higher proportion of White patients in non-TNBC group comparing to TNBC group (85% vs 72%, p = 0.0184). Although not statistically significant, there was a trend of higher proportion of Black patients in TNBC group comparing to non-TNBC group (21% vs 11%, p = 0.0515). The average tumor size of TNBC and non-TNBC was 2.2 cm (range, 0.7–28) and 1.8 cm (range, 0.9–11), respectively. TNBC had significantly larger tumor size and higher histologic and nuclear grade compared to non-TNBC **(** **Table 1** **)**. The incidence of LVI was similar in TNBC and non-TNBC, although TNBC had slightly higher rate of axillary lymph node metastases **(** **Table 1** **)**. The histologic sub-types of TNBC included: invasive ductal carcinoma not otherwise specified (IDC-NOS) (n = 218), metaplastic carcinoma (n = 5), pleomorphic lobular carcinoma (n = 1) and mixed ductal and pleomorphic lobular carcinoma (n = 2). Among the non-TNBC, 76 were IDC-NOS type. The remaining non-TNBC were invasive lobular carcinoma (n = 8), mixed ductal and lobular carcinoma (n = 3) and invasive mucinous carcinoma (n = 1).

**Table 1:** Clinicopathologic Features of TNBC versus non-TNBC.

	TNBC (n = 226)	non-TNBC (n = 88)	p value
Mean age (years) [range]	55 [54–57]	54 [51–57]	0.39
Race			
White	163 (72%)	75 (85%)	0.0184
Black	48 (21%)	10 (11%)	0.0515
Asian	13 (6%)	1 (1%)	0.1236
Other	2 (1%)	2 (2%)	0.3132
Mean tumor size (cm) [range]	2.2 [0.7–28]	1.8 [0.9–11]	0.04
Histologic grade			<0.00001
1	0 (0%)	0 (0%)	
2	9 (4%)	25 (28%)	
3	217 (96%)	62 (71%)	
Nuclear grade			<0.00001
1	0 (0%)	0 (0%)	
2	11 (5%)	46 (52%)	
3	215 (95%)	42 (48%)	
Lymphovascular invasion	83 (37%)	32 (36%)	0.53
Lymph node metastasis	129 (57%)	39 (44%)	0.044

Abbreviations: TNBC = triple negative breast carcinoma.

### Immunohistochemical analysis of MSLN expression

**Figure 1:**
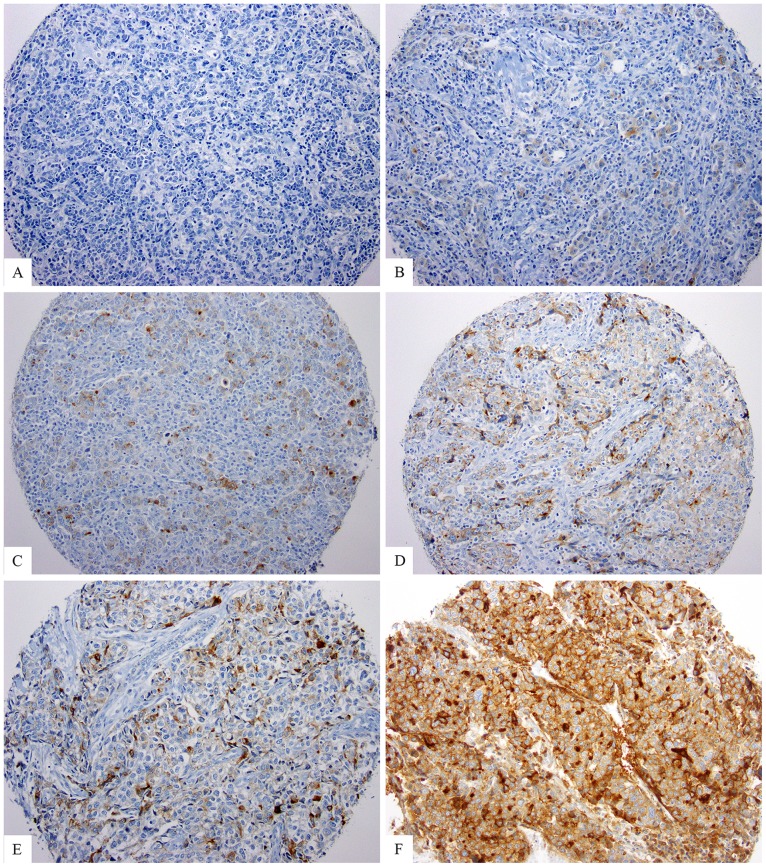
Examples of immunohistochemical staining for MSLN in TNBC. A and B) MSLN negative. A) No staining. B) Percentage score = 1 (1–50%), intensity score = 1 (weak), final score = 2. C–F) MSLN positive. C) Percentage score = 1 (1–50%), intensity score = 2 (moderate), final score = 3. D) Percentage score = 2 (>50%), intensity score = 1 (weak), final score = 3. E) Percentage score = 2 (>50%), intensity score = 2 (moderate), final score = 4. F) Percentage score = 2 (>50%), intensity score = 3 (strong), final score = 5.

Immunohistochemical stain for MSLN yielded uniform, strong cytoplasmic and membranous reactivity in the mesothelioma tumor cells used as positive control. Minimal background staining was detected in inflammatory cells or benign stroma. In the breast cancer specimens, MSLN showed variable staining intensity and percentage in the tumors cells (**Fig. 1** **,** **Table 2**). A significantly higher proportion of TNBC (82/226, 36%) showed MSLN score ≥3 than non-TNBC (14/88, 16%) (Fisher’s exact test, p = 0.0006) **(** **Tables 2** **and** **3** **)**.

**Table 2:** Distribution of MSLN scores in TNBC versus non-TNBC.

MSLN Score	0	1	2	3	4	5
TNBC (n = 226)	77 (34%)	28 (13%)	39 (17%)	43 (19%)	27 (12%)	12 (5%)
Non-TNBC (n = 88)	48 (55%)	15 (17%)	11 (13%)	6 (7%)	8 (9%)	0 (0%)

Abbreviations: MSLN = mesothelin; TNBC = triple negative breast carcinomas.

**Table 3:** MSLN correlates significantly with TNBC status.

	TNBC (n = 226)	Non-TNBC (n = 88)	ER/PR(+)/HER2(−)	ER/PR(+)/HER2(+)	p-value
MSLN (+)	82 (36%)	14 (16%)	10 (11%)	4 (5%)	*0.0006
MSLN (−)	144 (64%)	74 (84%)	57 (65%)	17 (19%)	

Abbreviations: MSLN = mesothelin; TNBC = triple negative breast carcinomas. *comparing TNBC with non-TNBC.

Among TNBC, MSLN was positive in 80 of 218 IDC-NOS and 2 out of 5 metaplastic carcinomas. MSLN expression correlated with slightly older age at diagnosis, but not with race, tumor size, histologic or nuclear grade, LVI or lymph node metastases **(** **Table 4** **)**. MSLN positivity also significantly correlated with basal keratins CK5/6 (56/80, 70%; p<0.00001) and CK14 (29/72, 40%; p = 0.017), but not with EGFR (57/78, 73%; p = 0.87) **(** **Table 5** **)**.

**Table 4:** MSLN expression and clinicopathological characteristics of TNBC.

	MSLN(+) (n = 82)	MSLN(−) (n = 144)	p value
Mean patient age (years)	58.0	54.0	0.043
Race			
White	58 (71%)	105 (73%)	0.7588
Black	21 (26%)	27 (19%)	0.2398
Asian	3 (4%)	10 (7%)	0.3845
Other	0	2 (1%)	0.5356
Mean tumor size (cm)	2.3	2.2	0.70
Histologic grade			0.49
1	0 (0%)	0 (0%)	
2	2 (2%)	7 (5%)	
3	80 (98%)	137 (95%)	
Nuclear grade			0.20
1	0 (0%)	0 (0%)	
2	2 (2%)	9 (6%)	
3	80 (98%)	135 (94%)	
Lymphovascular invasion	36 (44%)	47 (33%)	0.11
Lymph node(+)	40 (49%)	89 (62%)	0.07

Abbreviations: MSLN = mesothelin; TNBC = triple negative breast carcinoma.

**Table 5:** MSLN and basal markers expression in TNBC.

Basal marker	TNBC MSLN(+) (n = 82)	TNBC MSLN(−) (n = 144)	p value
CK5/6(+)	56/81 (69%)	50/142 (35%)	<0.0001
CK14(+)	29/72 (40%)	33/137 (24%)	0.017
CK5/6 and/or CK14(+)	63/76 (83%)	63/137 (46%)	<0.0001
EGFR(+)	57/78 (73%)	102/139 (73%)	0.88
CK5/6 and EGFR(+)	43/74 (58%)	53/134 (39%)	0.013
CK5/6 and/or EGFR(+)	57/78 (73%)	102/137 (75%)	0.82

Abbreviations: MSLN = mesothelin; TNBC = triple negative breast carcinomas. *CK5/6 status unknown for 3 cases, CK14 status unknown for 17 cases, EGFR status unknown for 9 cases.

Among non-TNBC, 67 cases were ER and/or PR positive and HER2 negative, 21 were ER and/or PR positive and HER2 positive. MSLN was positive in 13 out of 76 IDC-NOS and one mixed ductal and lobular carcinoma. No statistically difference in the prevalence of MSLN expression was observed between ER/PR positive, HER2 negative and ER/PR positive, HER2 positive tumors **(** **Table 3** **)**.

### Correlation with clinical outcome

The median follow-up time was 5.3 years (range 0.7–8.2). We observed a general trend towards increased frequency of distant metastases in patients with MSLN-positive TNBC, compared to patients with MSLN-negative TNBC and non-TNBC. Patients with MSLN-positive TNBC also had shorter interval to metastases and showed a greater propensity to develop brain metastasis **(** **Table 6** **)**.

**Table 6:** Correlation of MSLN expression and Distant Metastasis.

	TNBC MSLN(+)	TNBC MSLN(−)
Patients distant metastasis	16/70* (23%)	12/128* (9%)
Median interval to metastasis (month) [95% CI]	19.2 [13.5–24.9]	35.2 [23.8–46.6]
Site of metastases		
Bone	2 (13%)	2 (17%)
Brain	10 (63%)	4 (33%)
Liver	2 (13%)	2 (17%)
Lung	8 (50%)	4 (33%)
Multiple	6 (38%)	3 (25%)

Abbreviations: MSLN = mesothelin; TNBC = triple negative breast carcinomas. *Distant metastasis status unknown in 28 TNBC cases.

**Figure 2:**
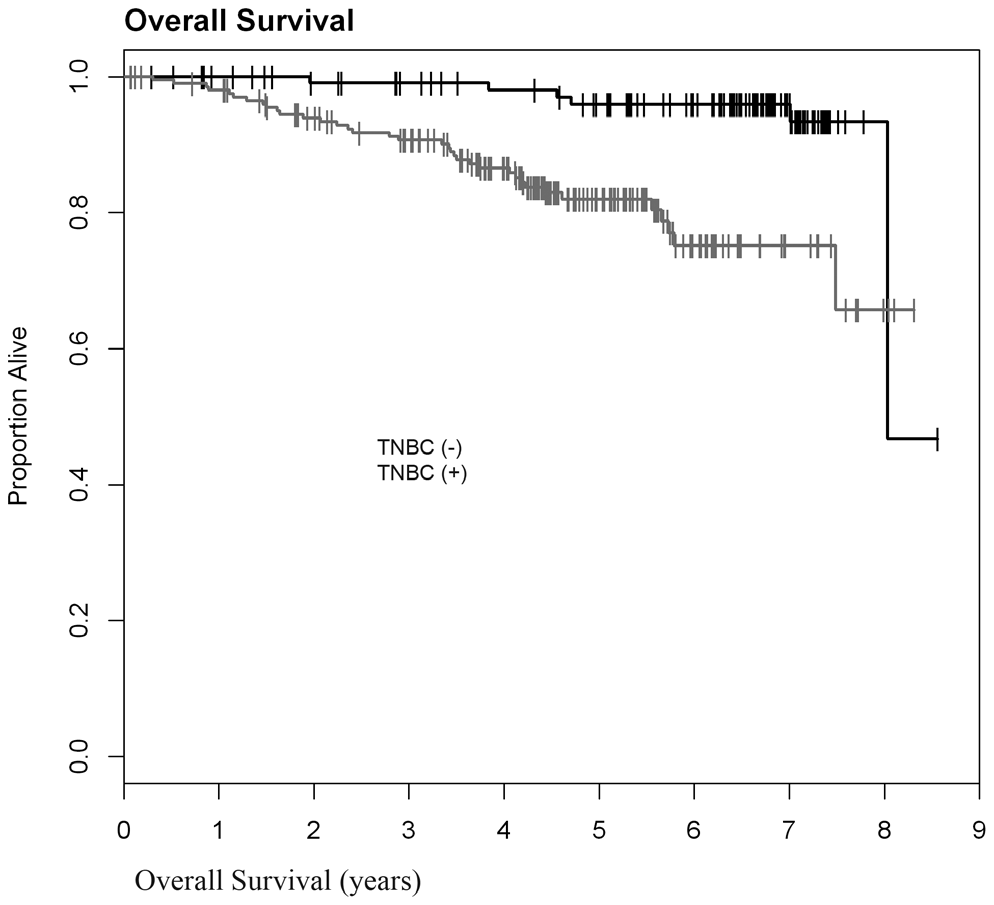
Decreased 5-Year overall survival in TNBC compared to non-TNBC (n = 314, p = 0.0001).

**Figure 3:**
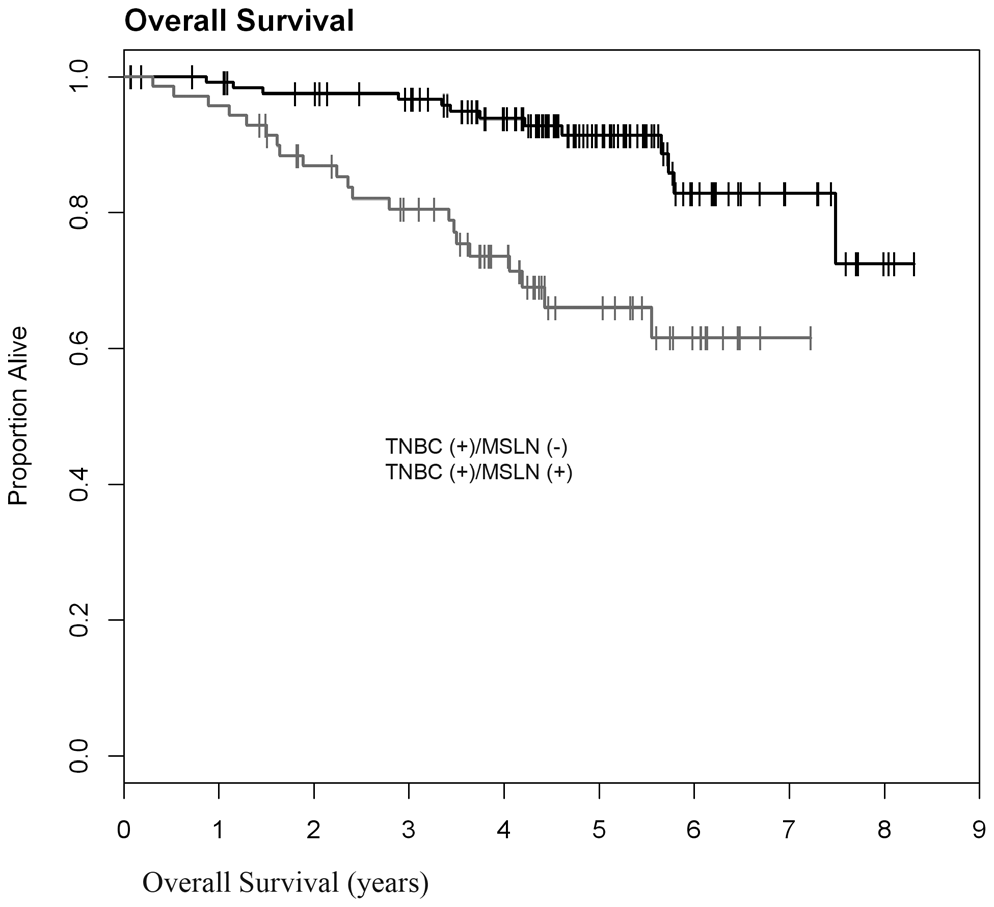
MSLN expression in TNBC correlates with significantly decreased overall survival (p<0.0001, n = 198).

**Figure 4:**
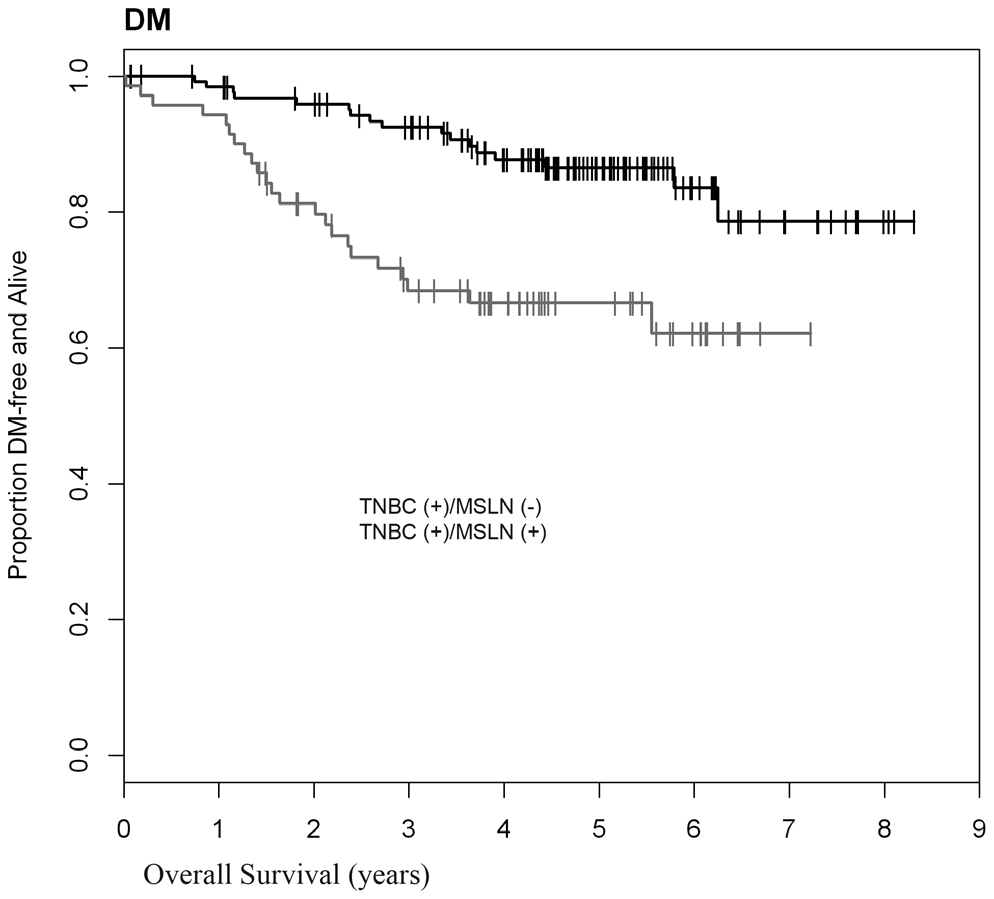
MSLN expression in TNBC correlates with significantly decreased disease-free survival (p = 0.0003, n = 198). *Distant metastasis (DM) status unknown in 28 TNBC cases.

**Figure 5:**
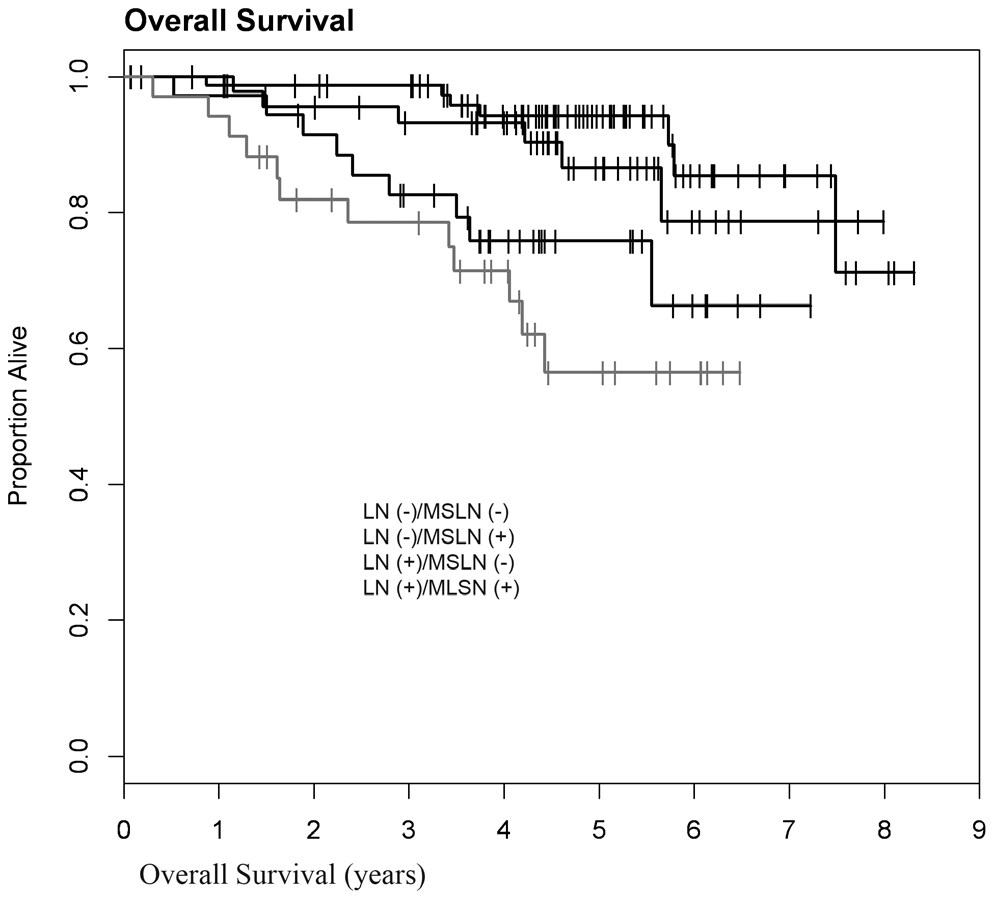
The difference in overall survival for MSLN(+) TNBC is independent of lymph node (LN) status (log rank p = 0.0003, n = 188).

**Figure 6:**
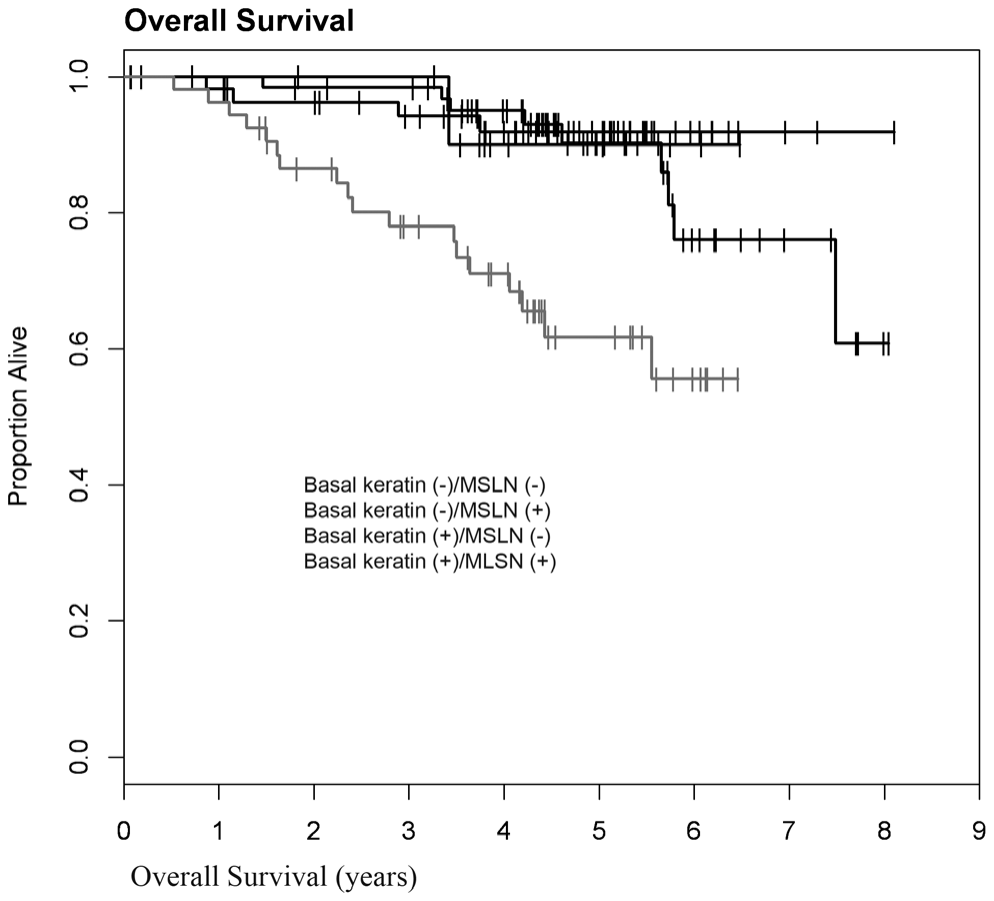
The difference in overall survival for MSLN(+) TNBC is independent of basal marker expression (log rank p = 0.0002, n = 188). Basal-keratin(+): CK5/6 and/or CK14 positive; basal-keratin(−): CK5/6(−) and CK14(−).

The 5-year Kaplan-Meier survival estimates confirmed that TNBC had significantly shorter overall probability of survival (0.82; 95% Cl: 0.75–0.87), compared to non-TNBC (0.959; 95% Cl: 0.895–0.984) **(** **Fig. 2** **)**. Nonetheless, among patients with TNBC, MSLN positivity correlated with significantly shorter OS (0.659; 95% Cl: 0.515–0.770 versus 0.913; 95%CI: 0.838–0.954) **(** **Fig. 3** **)**, and DFS (0.665; 95%CI: 0.536–0.766 versus 0.865; 95%CI: 0.785–0.916) **(** **Fig. 4** **)**. Node-positive and MSLN-positive TNBC fared the worst, with 5-year OS of 0.564 (95%CI: 0.348–0.733), compared to node-positive and MSLN-negative TNBC (0.865; 95%CI: 0.699–0.943), and to node-negative and MSLN-positive TNBC (0.758; 95%CI: 0.572–0.871) **(** **Fig. 5** **)**. Analysis of the survival data by log-rank test suggests that the negative survival impact of MSLN is independent of lymph node status (log rank test, p = 0.0003). Basal-keratin (CK5/6 and/or CK14) positive and MSLN-positive cases had significantly lower 5-year OS (0.617; 95%CI: 0.449–0.747) compared to all of the other groups **(** **Fig. 6** **)**. The survival impact of MSLN also appears independent of basal-like phenotype.

## Discussion

Most TNBC have an aggressive clinical course and do not respond to current therapies targeting ER, PR, and HER2. MSLN is a cell-surface antigen overexpressed in several human malignancies and constitutes a promising immunotherapy target [1]–[8], which could provide a much needed therapeutic option for patients with TNBC.

Several published studies have evaluated MSLN expression in different tumor types, using various scoring systems to quantify the immunohistochemical expression of MSLN. Argani et al [3] categorized any tumor with ≥1% staining of any intensity as “positive” for MSLN and any staining between 1%–25% as “focal”. Swierczyncki et al [4] also used similar cut-off percentage, but required “moderate to strong” labeling intensity. In their study, cases with “focal” staining and “positive” cases were combined together for statistical analysis [4]. Ho et al [6] did not explicitly specify an immunohistochemical cut-off score in their analysis. Frierson et al [8] scored MSLN immunoreactivity as absent, 1+ (<10% positive cells), 2+ (10%–50% positive cells), or 3+ (>50% positive cells). Tchou et al [46] evaluated 99 primary breast cancer samples by IHC and regarded as “positive” cases with MSLN staining in greater than 1% of tumor cells. The median proportion of cells positive for MSLN was 10% (range of 5–80%), with varied staining intensity [46]. Using these criteria, the authors reported MSLN overexpression in 67% (29/43) of TNBC, compared to only 3% and 4% of ER-positive and HER2-positive tumors respectively [46].

**Table 7** summarizes studies evaluating the prognostic significance of mesothelin expression by immunohistochemistry in a variety of adenocarcinomas [47]–[55]. Although there is no consensus on the scoring criteria of immunohistochemical staining for mesothelin, “high level” of mesothelin expression was significantly associated with worse outcome [48], [50], [52], [53], [55].

**Table 7:** Summary of published studies of MSLN immunohistochemical studies and correlation with survival.

Study	MSLN antibodyand dilution	Scoring methods	TMA vs wholetissue section	Cancer type	Correlation withsurvival
Yenet al [47]	clone 5B2,Promega, 1∶500	0: <5%; 1+: 5% to 50%; 2+: 51%to 75%; 3+: 76% to 95%; 4+: >95%	Wholetissue section	Ovarian serouscarcinoma	Related to favorableoverall survival
Einamaet al [48]	clone 5B2,Novocastra, 1∶50	High levels: >50% with any intensity,or moderate to strong intensity ofany percentage. Low level: <50%with weak intensity or absent	Wholetissue section	Pancreaticcancer	Co-expression ofmesothelin and CA-125(high levels for both) isassociated with poor prognosis
Babaet al [49]	clone HBME1, DAKO, 1∶50	0: <5%; 1∶5–50%; 2∶51–100%	Wholetissue section	Gastriccancer	Correlated withprolonged survival
Shimizuet al [50]	clone 5B2,Novocastra, 1∶20	Score = Staining intensity (0, 1, 2,3)×percentage. Cut-off was setat the medial score. High: >median. Low: <median	Wholetissue section	Pancreatic ductadenocarcinomas	Co-expression ofmesothelin and MUC16(high levels for both) isassociated with poorprognosis
Winteret al [51]	Vector Labs,1∶100	0: <10%; 1+: 11–25%; 2+:26–75%; 3+: >75%	Tissuemicroarray	Pancreatic adenocarcinomas	Significant predictors ofearly cancer-specific mortality
Kawamataet al [52]	clone 5B2,Novocastra, 1∶50	High levels: >50% with any intensity,or moderate to strong intensity ofany percentage. Low level: <50%with weak intensity or absent	Wholetissue section	Extrahepatic bile duct cancer	High-level expressionwas correlated with liver metastasis andpoor patient outcome
Einamaet al [53]	clone 5B2,Novocastra, 1∶50	Positive: >50% with any intensity,or moderate to strong intensity ofany percentage. Negative: <50%with weak intensity or absent	Wholetissue section	Gastriccancer	Poor prognosticfactor
Parinyanitikulet al [54]	clone 5B2, Novocastra, 1∶30	H score = staining intensity (0, 1, 2,3) ×percentage. Positive: H score >10	Tissuemicroarray	Triple negativebreast cancer	Mesothelin expression did notcorrelate with survival outcomes
Kachala et al[55]	clone 5B2,Vector lab, 1∶200	Sum score = Intensity (0, 1, 2, 3)+percentage(0: staining absent; 1∶1%–50%; or2∶51%–100%). High: sum score5. Low: sum score 0–4	Tissuemicroarray	Lung adenocarcinoma	High expressioncorrelates withworse survival
Currentstudy	clone 5B2,Vector lab, 1∶50	Sum score = Intensity (0, 1, 2,3)+percentage (0: staining absent;1∶1%–50%; or 2∶51%–100%).Positive: sum score >3	Tissuemicroarray	Triple negativebreast cancer	Correlates withworse survival

In our study of 226 TNBC treated at our institution we found MSLN to be overexpressed in 36% of cases. The rate of MSLN-positive TNBC in our study may appear relatively low compared to that reported by Tchou et al [46], because we regarded as MSLN-positive in only cases that showed substantial MSLN reactivity, including at least moderate staining intensity or if weak intensity in more than 50% of the tumor cells. Our scoring criteria is similar to that used in previous studies [48], [52], [53] demonstrating prognostic significance of mesothelin expression by immunohistochemistry in pancreatobiliary and gastric carcinomas. The use of a high cutoff of MSLN positivity, albeit arbitrarily selected, identifies cases that are more likely to be targetable to treatment with anti-MSLN. Interestingly, in our series, the use of strict criteria of MSLN- positivity identified carcinomas with significantly worse clinical behavior.

A recent study by Parinyanitikul and colleagues showed no correlation between MSLN expression and survival outcomes in triple negative breast carcinomas [54]. MSLN staining was quantified using an H-score that combined the percentage (0–100%) and intensity (1+, 2+, 3+). The H-score was calculated by multiplying the percentage of positive cells by a factor representing the intensity of immunoreactivity, with final score ranging between 0 and 300. An H-score of 10 was chosen as the threshold for MSLN positivity [54]. Using this scoring system, 37 (34%) of 109 TNBC were deemed MSLN positive [54], but MSLN expression in TNBC did not show prognostic significance. In contrast, we found that MSLN positive TNBC had significant worse prognosis. The difference between the study by Parinyanitikul et al and ours probably stems from the different criteria used for MSLN positivity. In addition, in the study by Parinyanitikul et al [54], TNBC was defined as ER and PR ≤5%, HER2 negative by IHC and or FISH. The different criteria used to assign ER and/or PR positivity in the study by Parinyanitikul et al [54] (<5%) and in our own (<1%) [44] may also account in part for the different results.

Over two-thirds of TNBC in our series demonstrated at least focal MSLN staining in at least 1% of tumor cells **(** **Table 2** **)**, a percentage similar to that reported by Tchou et al [46], but we documented higher rates of MSLN reactivity in non-TNBC (45% showing at least focal staining, and 16% with substantial MSLN expression in our study versus only 3% in a prior study [46]). However, in our series significantly more TNBC than non-TNBC were strongly MSLN-positive (82/226, 36% vs 14/88, 16%; p = 0.0006). Differences in proportion and intensity of staining could potentially be explained by differences in the choice of MSLN antibody dilution utilized, and definitive characterization of MSLN expression in non-TNBC requires further evaluation.

To the best of our knowledge, our series is the largest to date to assess MLSN expression in TNBC, allowing to further evaluate its correlation, or lack thereof, with basal immunophenotype. In our study, MSLN expression in TNBC correlated with basal cytokeratin expression but not with that of EGFR. Furthermore, MSLN expression was a predictor of worse outcome independent of basal immunophenotype.

In conclusion, MSLN, a cell surface antigen overexpressed in several malignancies, shows substantial expression in TNBC. Among TNBC, MSLN appears to be an independent prognostic marker associated with distant metastasis and worse survival. Patients with MSLN-positive TNBC could benefit from MSLN-targeted immuno therapies currently in development.
